# Innovative Advanced Materials for Energy Storage and Beyond: Synthesis, Characterisation and Applications

**DOI:** 10.3390/nano10091817

**Published:** 2020-09-11

**Authors:** Vijay Kumar Thakur

**Affiliations:** 1 Biorefining and Advanced Materials Research Center, Scotland’s Rural College (SRUC), Kings Buildings, Edinburgh EH9 3JG, UK; Vijay.Thakur@sruc.ac.uk; 2 Department of Mechanical Engineering, School of Engineering, Shiv Nadar University, Uttar Pradesh 201314, India

Recently, advanced materials have attracted considerable interest owing to their possible applications in different fields such as in catalysts, supercapacitors, capacitors, batteries and other energy storage systems [1,2,3]. Many of the 21st century’s advancing technologies, e.g., electric vehicles (and hybrids), portable electronic devices, and renewable energy systems, drive the demand for high-performance energy storage systems [4]. The increasing demand for processable, lightweight, flexible energy storage materials has motivated researchers from both academia and industry to develop and manufacture new materials that offer excellent properties depending on the targeted applications, including environmental applications [5,6]. Building upon the different potential of the advanced materials for several applications, this Special Issue has been aimed at presenting the current state-of-the-art in new advanced materials to address the various challenging issues researchers have been confronted with in this field for many applications, especially for energy storage. In this issue, we have featured 12 papers that include one excellent review “Sustainable Biomass Activated Carbons as Electrodes for Battery and Supercapacitors—A Mini-Review” and one communication article. In this Special Issue, we have covered the most recent advances that address novel and state-of-the-art topics from active researchers in innovative advanced materials and hybrid materials, concerning not only their synthesis, preparation and characterisation, but especially focusing on the applications of such materials with outstanding performances. This Special Issue has targeted readers from different disciplines.

Comprehensive and fundamental research has been published in this Special Issue, with the very first contribution from University of Cambridge researchers entitled “Non-Isothermal Crystallisation Kinetics of Carbon Black-Graphene-Based Multimodal-Polyethylene Nanocomposites”. In this work, Ahmad et al. have reported their findings on the carbon black-graphene reinforced High Density Polyethylene (HDPE) composites based on crystallisation kinetics [7]. In this work, the different types of composite materials were prepared using the varying ratio of the filler (carbon black/graphene) from 0.1 to 5 wt.% using the non-isothermal conditions. The graphene content along with the cooling rate was found to have a great impact on the crystallisation behaviour (the non-isothermal of the PE-g nanocomposites). It was found that the PE-g relative peak crystallisation temperature improved with the reduction in the cooling rate for a selected reinforcement (e.g., graphene content). At a specified cooling rate, it was found to increase progressively with an enhancement in the graphene concentration as well as transformation in the nucleation mechanism. It was concluded from the study that the polyethene (PE)-g nanocomposite’s non-isothermal crystallisation behaviour depends considerably on both the content of graphene and the cooling rate.

Cabello et al., in their work, have explored the usage of MgCl_2_ as an electrolyte to increase the Li_4_Ti_5_O_12_ (LTO) electrochemical performance as the novel cathode in next-generation Mg batteries [8]. Various compositions of the electrolyte were investigated to study the usage of LTO electrodes in Mg batteries. It was demonstrated in this study that the first discharge, as well as charge profile, exhibited a plateau among 0.4–0.3 V–1.35 V Mg^2+/^Mg_0_, respectively, using a solution of 0.5 M Mg(TFSI)_2_ + 0.13 M MgCl_2_·6H_2_O in DME. Subsequently, at 0.6–0.5 V, the potential was sustained on further discharges. The authors reported to have attained 175 and 290 mAh g^−1^ capacities, corresponding to the establishment of Mg_1.5_ L_i4_Ti_5_O_12_, and Mg_2.5_ Li_4_Ti_5_O_12_, respectively. The authors also emphasised that further work is needed to advance the LTO capacity retention over a huge number of cycles. In another interesting work, Dong and co-workers have reported their findings on enhancing the Garnet Li_7_La_3_Zr_2_O_12_ (as promising electrolyte) ionic conductivity via spark plasma sintering and dual substitution [9]. In this work, the authors have explored the use of Ta for Zr and Mg for Li as the dual substitution strategy to analyse the structure and performance of garnet Li_7_La_3_Zr_2_O_12_. The garnet, having an arrangement of Li_6.5_ Mg_0.05_La_3_Zr_1.6_Ta_0.4_O_12_, exhibited a single cubic phase with an ionic conductivity of 6.1 × 10−4 S cm^−1^, which was better in comparison to the pristine Li_6.6_ La_3_Zr_1.6_ Ta_0.4_ O_12_. It was concluded from the study that the spark plasma sintering (SPS) densified the garnets and enhanced their ionic conductivities [9].

Lee et al., in their work, reported on the development of activated carbons from thermoplastic precursors and used them for energy storage applications [10]. The low-density polyethene (LDPE) was used to prepare the activated carbons (PE-AC) as novel electrode materials for an electric double-layer capacitor (EDLC). Methods such as carbonisation, cross-linking, and subsequent activation under different conditions were used. Different characterisation techniques, such as Cs-corrected field-emission transmission electron microscope, field-emission scanning electron microscope, and X-ray diffraction analysis, were used to analyse the surface morphologies as well as the structural characteristics. Brunauer–Emmett–Teller, Barrett–Joyner–Halenda equations and nonlocal density functional theory were used to confirm and characterise the nitrogen adsorption isotherm-desorption. The research demonstrated that with the enhancement in the activation time, total pore volume and the specific surface area and of the activated samples increased. The total pore volume (0.86 cm^3^/g), specific surface area (1600 m^2^/g), and mesopore volume (0.3 cm^3^/g) of the PE-AC were observed and the PE-AC demonstrated a higher by 35% mesopore volume ratio. It was concluded from the study that the LDPE’s structural characteristics and the activation conditions have been found to affect the electrode materials performance [10].

In addition to the Li-ion batteries, recently there has been a great thrust on exploring other alternatives. In this direction, ceramic fluorine ion conductors that exhibit much better ionic conductivity in comparison to others have recently emerged as the most promising materials and are currently being explored in fluorine-ion batteries (FIBs) as a new class of solid-state electrolytes. In the same line, we have an interesting article on fluoride-ion batteries. In this work, authors have reported on the nanocrystalline La_0.9_ Ba_0.1_ F_2.9_ electrochemical stabilities against metal electrodes [11]. They analysed the electrochemical stability of numerous metal electrodes having the potential to act as current collector materials in the state-of-the-art fluorine-ion batteries. It was concluded from the study that most of the tested metals were not in stable contact with the La_0.9_ Ba_0.1_ F_2.9_ and the FIBs, hence, the selection of current collectors will be an important issue [11]. Siwal et al. have, on the other hand, reported on the usage of graphitic carbon nitride (gCN) as an innovative support material for the synthesis of copper–manganese alloy (CuMnO_2_) [12]. Different characterisation techniques such as optical and spectroscopic were used to confirm the formation of CuMnO_2_-gCN. The synthesised catalyst in the alkaline media was used as the energy storage material that demonstrated decent catalytic behaviour in supercapacitors applications. For example, the CuMnO_2_-gCN modified GCE demonstrated improved electrochemical performance in comparison to that of the Cu_2_O-gCN electrode [12].

Tesfaye et al. have reported their work on the development of li-ion microbatteries [13]. In this work, the atomic layer deposition (ALD) method was used to decorate MoS_2_ in a homogenous way. Different techniques, such as energy dispersive X-ray spectroscopy, scanning transmission electron microscopy, chronopotentiometry and X-ray photoelectron spectroscopy, were used to investigate the electrochemical performance, morphology and structure of the Al_2_O_3_/MoS_2_/Al_2_O_3_-decorated TiO_2_ nanotube layers (TNTs). It was concluded from the study that TNTs decorated using Al_2_O_3_/MoS_2_/Al_2_O_3_, demonstrated as three times higher, and deliver aerial capacity in comparison to MoS_2_-decorated TNTs [13]. In another work, Al-Shehri et al. have reported their work on the design and development of nano-catalysts where authors have used noble metal nanoparticles to support mesoporous silica [14]. Authors were able to incorporate the M_0_ nanoparticles of (Pt Rh, or Au, Pd) having a 5–10 nm average size into the siliceous TUD-1 mesoporous material employing a sol-gel method that was surfactant-free. The CO oxidation was used at a low temperature to analyse the catalytic performance of synthesised nano-catalysts as a model system. The Au-TUD-1 catalyst among all the studied catalysts was found to demonstrate the highest catalytic performance followed by Pt-TUD-1 and Pd-TUD-1. On the other hand, at a higher temperature, the Rh-TUD-1 displayed the lowermost activity. It was reported that the developed catalysts exhibit salient features for promising applications in several fields, such as respiratory/escape masks for removing gases, air purification, devices for self-rescue breathing, refuge chambers, and numerous others [14].

Among various types of materials being used for energy storage in Li-Ion batteries, MXenes and 2D transition metal dichalcogenides are rapidly emerging as promising candidates for several applications including batteries and supercapacitors [15]. MXene is a new class of nanomaterials that were first described in 2011. MXene is generally derived from the ternary structured MAX phases and contain metal carbides, carbonitrides and nitrides. The latter currently comprise over 60 known phases. In this Special Issue, in an interesting paper, Nguyen et al. have reported their studies on the synthesis of new materials such as W_2_C/WS_2_ alloy nanoflowers (NF) to be used as an anode in lithium-ion storage [16]. A well-established facile hydrothermal methodology was used to fabricate W_2_C/WS_2_ NFs at low temperature. The authors were able to control the particle size in the range of 200 nm^–1^ μm and these NFs demonstrated hexagonal structures of W_2_C and WS_2_ along with high purity. Subsequently, these NF alloys were used in lithium-ion batteries (LIBs) as anode materials. It was concluded from the study that the prepared W_2_C/WS_2_ alloy NFs showed great potential for applications in energy storage as well as conversion [16].

Along with lithium-ion batteries and supercapacitors, solar cells are another class of requisite energy source that are being explored as renewable alternatives to petrochemical resources. The unique properties of the solar cells include that they never use fossil fuels and also have zero contribution to greenhouse gases. However, to obtain the solar cell with the desired efficiency, the materials used in them should exhibit appropriate sunlight absorbing efficiency and ability to convert them to electricity. One of the best solutions for this is that photovoltaic power conversion efficiency can be realised through the combination of dissimilar solar cells with complementary absorption ranges.

Jiang et al., in their interesting work, have reported on the solution processing of CdTe nanocrystal (NC) solar cells [17]. In this work authors have reported on the development of 2,2′,7,7′-tetrakis [N, N-di(4-methoxyphenyl) amino]-9,9′-spirobifluorene (Spiro) as a hole transfer layer (HTL) for solution-processed CdTe NC solar cells. It was reported from the study that through the annealing treatment there was an increment in the hole mobility as well as conductivity of the NC solar cells having Spiro HTL. With the annealing temperature in the range of 100–130 °C, simultaneous improvements were reported for CdTe NC solar cells in Voc, Jsc, and fill factor (FF). It was concluded from the study that the Jsc, Voc, and power conversion efficiency (PCE) of the developed NC solar cells increase simultaneously because of the reduced contact resistance as well as an improved built-in electric field [17].

In another interesting study, Llusco et al. have reported their findings on “the kinetic and thermodynamic Studies on Synthesis of Mg-doped LiMn_2_O_4_ Nanoparticles” [18]. In this work, different types of nanoparticles were synthesised using an ultrasound-assisted Pechini-type sol-gel process and the impact of Mg doping amount on thermal decomposition of the prepared precursors was investigated in detail. In this work, four types of thermal decomposition zones were well-defined using the synthesis precursors mass-loss profiles such as (i) dehydration, (2) polymeric matrix decomposition, (3) carbonate decomposition and formation of manganese oxide spinel, and (4) manganese oxide spinel decomposition. In this work, the polymeric matrix’s thermal disintegration was recognised as the key zone encompassing fundamental reactions initiating LiMn_2_O_4_ spinel phase formation. Authors have also mentioned plans on studying the electrochemical properties of the synthesised materials in the follow-up work [18].

Lignocellulosic biomass-based materials, such as natural cellulosic fibres, straw, plants and wood, represent some of the most biorenewable raw materials for the development of numerous chemicals and materials [19,20,21]. Indeed, the development of high-value products from different biomass has become very necessary to advance the commercial sustainability and viability of future biomaterials and bioenergy processes [22]. In Biorefinery, generally, the carbohydrate fraction of the lignocellulose is converted into higher-quality products, whereas the residual lignin and other materials are discarded/burned. However, the different components of any biomass contain different components such as nanocrystal cellulose, hemicellulose and lignin that can be converted into valuable materials, for example, as electrode materials in battery and supercapacitors [23]. In this Special Issue, dos Reis et al., in their detailed review article, have summarised the recent developments on the usage of different types of biomass as electrode materials in batteries and supercapacitors in energy storage application [23]. Various pyrolysis and experimental conditions were described in detail for the production of biomass-derived carbon electrodes (CEs). It was concluded in this study that the biomass-based carbon materials represent a “sustainable way” for the uprising energy storage industry. A different challenge that one faces during these carbon electrode (CE) syntheses was also summarised [23].

To summarise, this Special Issue covers the most relevant advanced materials, such as sustainable carbonaceous materials for a wide range of energy storage applications, including batteries supercapacitors, solar cells and beyond. It is also believed that this Special Issue will provide new directions on advanced applications of different classes of advanced and functional materials.
